# A simple and efficient method for betalain quantification in *RUBY*-expressing plant samples

**DOI:** 10.3389/fpls.2024.1449409

**Published:** 2024-09-18

**Authors:** Dibyajyoti Pramanik, Keunsub Lee, Kan Wang

**Affiliations:** ^1^ Department of Agronomy, Iowa State University, Ames, IA, United States; ^2^ Crop Bioengineering Center, Iowa State University, Ames, IA, United States

**Keywords:** agroinfiltration, betalain, HPLC, marker gene, *Nicotiana benthamiana*, spectrophotometer, transgenic maize, transient transformation

## Abstract

The *RUBY* reporter system has demonstrated great potential as a visible marker to monitor gene expression in both transiently and stably transformed plant tissues. Ectopic expression of the *RUBY* reporter leads to bright red pigmentation in plant tissues that do not naturally accumulate betalain. Unlike traditional visual markers such as β-glucuronidase (GUS), luciferase (LUC), and various fluorescent proteins, the *RUBY* reporter system does not require sample sacrifice or special equipment for visualizing the gene expression. However, a robust quantitative analysis method for betalain content has been lacking, limiting accurate comparative analyses. In this work, we present a simple and rapid protocol for quantitative evaluation of *RUBY* expression in transgenic plant tissues. Using this method, we demonstrate that differential *RUBY* expression can be quantified in transiently transformed leaf tissues, such as agroinfiltrated *Nicotiana benthamiana* leaves, and in stable transgenic maize tissues, including seeds, leaves, and roots. We found that grinding fresh tissues with a hand grinder and plastic pestle, without the use of liquid nitrogen, is an effective method for rapid betalain extraction. Betalain contents estimated by spectrophotometric and High-Performance Liquid Chromatography (HPLC) analyses were highly consistent, validating that our rapid betalain extraction and quantification method is suitable for comparative analysis. In addition, betalain content was strongly correlated with *RUBY* expression level in agroinfiltrated *N. benthamiana* leaves, suggesting that our method can be useful for monitoring transient transformation efficiency in plants. Using our rapid protocol, we quantified varying levels of betalain pigment in *N. benthamiana* leaves, ranging from 110 to 1066 mg/kg of tissue, and in maize samples, ranging from 15.3 to 1028.7 mg/kg of tissue. This method is expected to streamline comparative studies in plants, providing valuable insights into the effectiveness of various promoters, enhancers, or other regulatory elements used in transgenic constructs.

## 1 Introduction

Reporter genes provide essential tools to study various aspects of biological processes. Diverse genetically encodable reporters have been developed and tested to monitor transgene expression, subcellular localization, protein stability, and stress-related responses (Chiu et al., 1996). Among them, the most used reporters are the green fluorescent protein (GFP) and its derivatives, such as RFP, CFP, and YFP (Chudakov et al., 2010). Additionally, β-glucuronidase (GUS) and Luciferase (LUC) are widely used reporters in plant research. These reporters have been very useful for decades but have some significant limitations. The fluorescent proteins require sophisticated instruments to visualize the fluorescent signals. Special light sources are needed to generate detectable signals, and usually, a microscope equipped with specific filter sets is used to visualize the fluorescence signals. GUS and LUC require expensive substrates X-Gluc (5-bromo-4-chloro-3-indolyl-β-D-glucuronic acid) and luciferin, respectively. More importantly, GUS-staining involves the sacrifice of precious transgenic plant tissues, whereas luciferase requires a special camera for visualization (Yuan et al., 2021).

Recently, natural pigment-based visual reporter systems have been developed for easy visualization of transgene expression without special equipment or sample pretreatments. An anthocyanin-based visual reporter system was tested for gene functional analysis and other applications (Fan et al., 2020; Xing et al., 2023; Grützner et al., 2024). Anthocyanin is a pigment commonly found in plants. However, recent reports suggested that anthocyanin synthesis is regulated by MYB and basic HELIX-LOOP-HELIX (bHLH) transcription factors that can interfere with other cellular processes and alter plant fitness (Fan et al., 2020).

**Figure 1:**
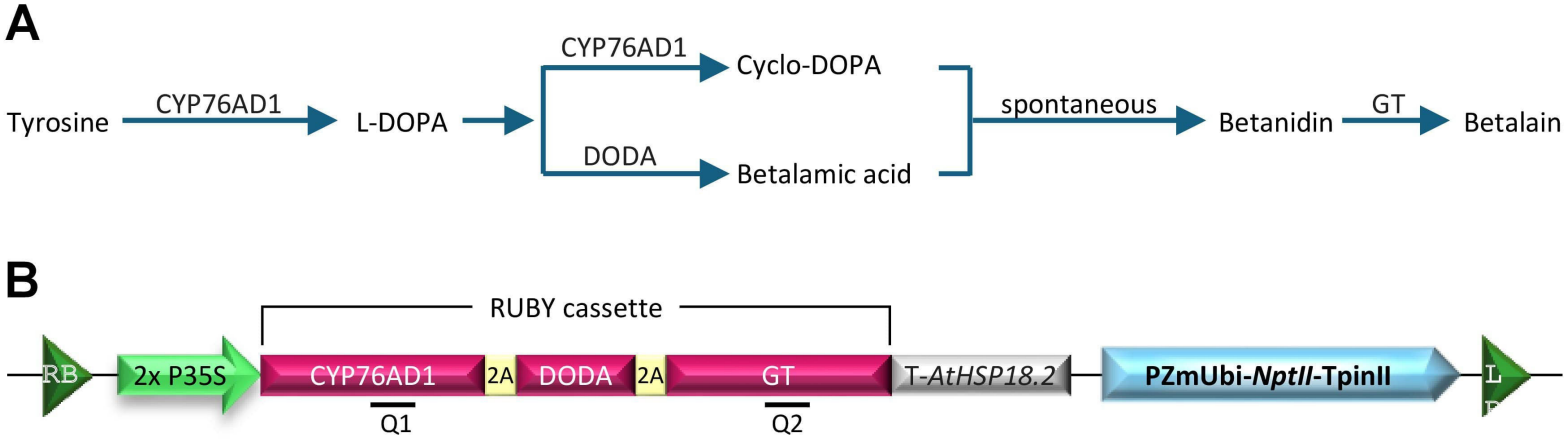
**(A)** Schematic representation of betalain biosynthesis pathway in plants. CYP76AD1 is a cytochrome P450 enzyme that catalyzes the conversion of tyrosine to L-DOPA (L-3,4-dihydroxyphenylalanine). DODA (L-DOPA 4,5-dioxygenase) cleaves L-DOPA to generate betalamic acid, whereas CYP76AD1 oxidizes it to cyclo-DOPA. Betalamic acid and cyclo-DOPA spontaneously combine to generate betanidin, which is further converted into betalain by glucosyl transferase (GT). **(B)** Schematic drawing of the T-DNA region of pCBL101-RUBY construct. 2×P35S, double Cauliflower Mosaic Virus (CaMV) 35S promoter; T-AtHSP18.2, *Arabidopsis thaliana HSP18.2* gene terminator; 2A, self-cleaving peptide P2A; PZmUbi-NptII-TpinII, a plant-selectable marker *neomycin phosphotransferase* II (*nptII*) gene expressed by maize ubiquitin gene promoter (PZmUbi) and a potato proteinase inhibitor II gene terminator (TpinII); Q1, RT-PCR fragment for *CYP76AD1* (positions 838 to 997); Q2, RT-PCR fragment for *GT* (positions 3502 to 3746); RB, right T-DNA border; LB, left T-DNA border.

Another pigment-based reporter, *RUBY*, was developed by He et al. (2020). This reporter system can be monitored in a noninvasive, continuous, and cost-effective manner. *RUBY* converts tyrosine into betalain, a significant natural pigment produced in plants. Accumulation of such pigment resulted in a bright red color predominantly found in beetroots, dragon fruit, leafy amaranth, and Swiss chard (Choo et al., 2019). The betalain biosynthesis pathway has been well studied (Timoneda et al., 2019; Tossi et al., 2021). The pathway relies on three enzymatic reactions to convert tyrosine into betalain (**Figure 1A**). Tyrosine is first converted to L-3,4-dihydroxyphenylalanine (L-DOPA), catalyzed by the enzyme P450 oxygenase CYP76AD1. L-DOPA can be further oxidized into cyclo-DOPA by CYP76AD1. Alternatively, L-DOPA is catalyzed by L-DOPA 4,5-dioxygenase (DODA) into betalamic acid, which is spontaneously condensed with cyclo-DOPA into betanidin. Lastly, betanidin is converted into the colorful pigment betalain by a glucosyltransferase (GT) (Polturak et al., 2016).

The *RUBY* reporter was produced by combining the open reading frames of the three key genes, *CYP76AD1*, *DODA*, and *GT*, separated by the self-cleaving peptide P2A under a single promoter such as double Cauliflower Mosaic Virus 35S promoter (2x P35S) and Arabidopsis *HSP18.2* terminator (**Figure 1B**; He et al., 2020). After translation, the P2A self-cleaving peptides are processed, allowing the individual enzymes to separate and carry out betalain biosynthesis. Ectopic *RUBY* expression and betalain accumulation were reported in various plant species, including tomatoes, potato, eggplant (Polturak et al., 2017), rice (Tian et al., 2020; He et al., 2020), Arabidopsis (He et al., 2020; Yu et al., 2023), maize (Lee et al., 2023; Wang et al., 2023), *Nicotiana benthamiana* (Chen et al., 2024; Yu et al., 2023; Jogam et al., 2024; Neubauer et al., 2024), *Citrus aurantifolia* (Thilmony et al., 2022), bamboo (Chen et al., 2023; Sun et al., 2023), carrot (Deng et al., 2023; Deng et al., 2023), *Plukenetia volubilis* (Yu et al., 2023), and cotton (Ge et al., 2023). Overall, the *RUBY* has been very useful in monitoring the plant transformation process and transgene segregation in the subsequent generations. While tissue-specific betalain accumulation was observed with inducible or tissue-specific promoters (Yang et al., 2023; Liu et al., 2024), just like other reporter systems, the *RUBY* reporter also has some limitations. For instance, *RUBY* cannot be used for intracellular protein localization or trafficking studies because betalains are water-soluble and accumulate in vacuoles (Solovchenko et al., 2019).

While differential betalain accumulation can be observed with the naked eye, quantitative measurement offers valuable tools for precisely monitoring transgene delivery and expression in various plant tissues (He et al., 2020). Beyond visual phenotyping, quantitatively estimating betalain content in plant tissues is highly beneficial for comparative studies. It enables statistical analysis to identify significant differences between samples and treatments. In addition, quantitative analysis allows data normalization to internal or external controls, reducing sample variability and thus enhancing reliability and reproducibility. Quantifying betalain from *RUBY*-expressing plant tissues involves three major steps: tissue homogenization, pigment extraction, and quantification. In the laboratory practice, liquid nitrogen was applied for tissue homogenization, followed by pigment extraction using an extraction solution (ES). Different ES was tested by diverse research groups such as methanol buffer (50% methanol, 1 mM ascorbic acid, 0.5% formic acid; Grützner et al., 2021), methanol: chloroform: H_2_O [1:2:1] (Chang et al., 2021), and H_2_O only (Tabara et al., 2024). Additionally, Liu et al. (2024) reported a serial extraction method in which a total of seven steps were used for extraction, including 40% methanol and 50 mM sodium ascorbate, followed by organic reagents (petroleum ether, acetone, trichloromethane, ethyl acetate, benzene, and cyclohexane) and chloroform. During the sample extraction, other natural plant pigments were released in the solution. Hence, extracted samples can be further processed and analyzed to quantify betalain by spectrophotometric methods (Cai and Corke, 1999; Grützner et al., 2021) or high-performance liquid chromatography (HPLC) (Castellanos-Santiago and Yahia, 2008). Pigment detection using mass spectrometry along with HPLC can measure the accurate pigment concentration in the tested solution. However, such techniques increase sample processing time and cost.

Here, we present a simple and robust method for quantifying betalain content in plant tissues, including its isolation from agroinfiltrated *N. benthamiana* leaves and stable transgenic maize seed, leaf, and root tissues. This method can be readily adopted for comparative studies requiring quantitative measurement of *RUBY* expression in transiently or stably transformed plant tissues.

## 2 Materials and equipment

### 2.1 Plant material, constructs, and *Agrobacterium* strains

*Agrobacterium tumefaciens* strain LBA4404Thy-: It is an auxotrophic thymidylate synthase gene (*thyA*) mutant strain of LBA4404 (Ranch et al., 2012; Anand et al., 2018). The strain was kindly provided by Dr. William Gordon-Kamm of Corteva Agriscience.

RUBY reporter plasmid pCBL101-RUBY (Addgene #199723): It includes Cauliflower Mosaic Virus 35S promoter (P35S) to drive the expression of three enzymes that sequentially convert tyrosine into a purple pigment betalain (He et al., 2020) (**Figure 1B**).

Ternary helper plasmid pKL2299 (Addgene #186332): It carries extra copies of the *Agrobacterium* virulence (*vir*) genes from the Ti plasmid pTiBo542 to improve plant transformation frequencies (Kang et al., 2022). pKL2299 has the RK2 origin of replication (ORI) and can be compatible with a binary vector plasmid that contains a pVS1 ORI.

*Nicotiana benthamiana* wild-type plants: seeds from lab stock or can be purchased commercially, and plants were grown according to Sainsbury and Lomonossoff (2008).

Maize inbred B104 seed can be obtained from the USDA-ARS Germplasm Resources Information Network (GRIN; https://www.ars-grin.gov/).

*RUBY*-expressing transgenic maize B104 samples: seeds from lab stock produced by Lee et al. (2023) and seedlings were germinated according to Kang et al. (2022).

### 2.2 Betalain extraction supplies

- 1.5 mL and 2 mL microcentrifuge tubes.
- 1600 MiniG Automated tissue homogenizer (SPEX Sample Prep, NJ, USA).
- 2.0 mm Zirconium oxide beads (Next Advance Inc, NY, USA).
- 96-well microplate (Cat # M33089; Thermo Fisher Scientific, NH, USA).
- Chloroform.
- Electric hand drill.
- Extraction Solution: Methanol: dH_2_O (1:1, v/v).
- Liquid nitrogen.
- Methanol.
- Mortar and pestle.
- Plastic pellet pestles (Cat # 12-141-367, Thermo Fisher Scientific, NH, USA).
- Scalpel and blades.
- Spatula.
- SpectraMax^®^ iD3 Multi-Mode Microplate Reader (Molecular Devices, LLC, CA, USA).
- Weighing balance (1-100 mg scale).

## 3 Methods

### 3.1 Plant sample collection, preparation, and betalain extraction

#### 3.1.1 Agroinfiltrated *Nicotiana benthamiana* leaves (2 days after infiltration)

Agroinfiltration experiments were performed using the protocol described by Sainsbury and Lomonossoff (2008).

##### 3.1.1 A Tissue grinding using liquid N_2_

A1. Label 2 mL microcentrifuge tubes, add one zirconium bead to each tube, and measure and record the weight.
A2. Precisely cut each infiltrated leaf area with scissors, transfer it to the 2 mL tube described above, record the weight again, and put the tube with tissue into liquid nitrogen.
A3. Calculate the fresh weight of each sample tissue by subtracting the weight of the empty tube plus the bead from the total weight of the tube with the leaf sample.
A4. Samples can be processed immediately or kept at – 80°C until analysis.
A5. Grind leaf tissue by placing the tube prepared in step A2 in a tissue homogenizer such as 1600 MiniG® (SPEX Sample Prep, Metuchen, NJ, USA), for 30 s at 1200 strokes/min at freezing temperature.
A6. Add 10 volumes of Extraction Solution (ES) per mg of fresh leaf tissue into each tube (e.g., 100 µL of ES for 10 mg tissue) for betalain extraction at room temperature.

##### 3.1.1 B Tissue grinding in ES

B1. Label 1.5 mL microcentrifuge tubes and measure the weight of each empty tube.
B2. Precisely cut the infiltrated leaf area with scissors and add it into each 1.5 mL tube.
B3. Record the weight before and after the sampling to calculate the amount of the tissue sample.
B4. Samples can be processed immediately or kept at 4°C for 1-2 days or kept at – 80°C until analysis.
B5. Add 10 volumes of ES per mg of fresh leaf tissue into each tube (e.g., 200 µL of ES for 20 mg tissue) for betalain extraction.
B6. Grind leaf tissue in ES using a hand grinder (such as an electric drill) with a plastic pestle. Homogenize the leaf tissue for 30-60 s at room temperature.

##### 3.1.1 C Betalain extraction

C1. After tissue grinding, water-soluble betalain is released into ES, producing pigmented leaf extract.
C2. Vortex the tubes for 30 s to thoroughly mix the ground leaf tissue with ES.
C3. Centrifuge the tubes at 21300 × g for 2-5 min at room temperature (RT) to separate the supernatant.
C4. Transfer the supernatant using a pipette into a new 1.5 mL microcentrifuge tube without disturbing the pellet.

#### 3.1.2 Maize seeds

1. Label 1.5 mL microcentrifuge tubes and measure the weight of each empty tube.
2. Freeze each maize seed using liquid N_2_ and grind it using a clean mortar and pestle.
3. Transfer ground maize seed powder using a clean spatula into the labeled 1.5 mL microcentrifuge tube.
4. Weigh the tubes after the sampling to measure the amount of each sample.
5. Add 10 volumes of ES per mg of seed powder into each tube (e.g., 500 µL of ES for 50 mg powder) for betalain extraction.
6. Vortex the tubes for 1-2 min to thoroughly mix the ground tissue with ES.
7. Centrifuge the tubes at 21300 × g for 2-5 min at RT to separate the supernatant.
8. Carefully transfer the supernatant using a pipette into a new 1.5 mL microcentrifuge tube without disturbing the pellet.

#### 3.1.3 Maize leaf

1. Label 1.5 mL microcentrifuge tubes and measure the weight of each empty tube.
2. Harvest wild-type and transgenic maize seedling leaf samples and process them individually.
3. Slice the leaves into 1-2 cm pieces using a clean, sharp scalpel blade.
4. Transfer 1-2 randomly selected pieces into the labeled 1.5 mL tube and measure/record the weight after the sampling to calculate the tissue weight.
5. Leaf samples can be processed immediately or stored at 4°C for 1-2 days or kept at - 80°C for long-term storage.
6. Add 10 volumes of ES per mg of fresh leaf tissue into each tube (e.g., 200 µL of ES for 20 mg tissue) for betalain extraction.
7. Grind leaf tissue in ES using a hand grinder (such as an electric drill) with a plastic pestle. Homogenize the leaf tissue for 1-2 min until the leaf sample turns into small debris or clumps.
8. Vortex the tubes for 1 min to thoroughly mix the ground leaf tissue with ES.
9. Centrifuge the tubes at 21300 × g for 5 min at RT to separate the supernatant.
10. Carefully transfer the supernatant using a pipette into a new 1.5 mL microcentrifuge tube without disturbing the pellet.

#### 3.1.4 Maize root

1. Label 1.5 mL microcentrifuge tubes and measure/record the weight of each empty tube.
2. Harvest wild-type and transgenic maize seedling root samples and process them individually.
3. Cut the roots into 2-3 cm segments using a clean, sharp scalpel blade.
4. Transfer the root segments into the labeled 1.5 mL tube and measure/record the weight to calculate the tissue weight.
5. Root samples can be processed immediately or stored at 4°C for 1-2 days or kept at – 80°C for long-term storage.
6. Add 10 volumes of ES per mg of fresh root tissue into each tube (e.g., 500 µL of ES for 50 mg tissue) for betalain extraction.
7. Grind root tissue in ES using a hand grinder (such as an electric drill) with a plastic pestle. Homogenize the root tissue for 2-3 min until the root sample turns into small debris or clumps.
8. Vortex the tubes for 1 min and centrifuge at 21300 × g for 5 min at RT.
9. Carefully transfer the supernatant using a pipette into a new 1.5 mL microcentrifuge tube without disturbing the pellet.

Note: *If cell debris is found in the supernatant, repeat the centrifugation step to prevent background noise.*

### 3.2 Betalain content measurement and analysis

It is recommended that betalain content is measured on the same day of extraction. Samples can be stored at 4°C for up to one week. Betalain content can be measured using a spectrophotometric analysis as previously described (Cai and Corke, 1999; Grützner et al., 2021):


$$\begin{array}{c} Betalain\;Content\;\left(mg/kg\;fresh\;tissue\right)\\ =[(A\times DF\times MW\times 1,000)/(\epsilon \times L)] \end{array}$$

Where:

- A, the absorption value at 538 nm.
- DF*, the dilution factor, 10 × sample dilution ratio.
- MW, the molecular weight of betalain, 550 g/mol.
- ϵ, the molar extinction coefficient, 60,000 L/mol·cm in H_2_O.
- L**, the path length, 1 cm for 1 mL cuvette and 0.2643 cm for a 96-well plate.

*The final DF is 10 × sample dilution ratio, because a 10× ES per mg tissue (w/v) is used during the betalain extraction step.

**The path length of 100 µL of sample solution in a 96-well plate is calculated as follows:


$$\begin{array}{c} L=V/A=V/\pi \times r^{2}=100\;mm^{3}/37.82\;mm^{2}=2.643573\;mm\\ =0.2643573\;cm \end{array}$$

The volume V of 100 µL is equivalent to 100 mm^3^. The diameter of each well of a 96-well plate is 6.94 mm. Thus, the radius r is 3.47 mm (6.94/2 = 3.47 mm). The area A of each well is π × r^2^ = 3.141592 × 3.47 mm × 3.47 mm = 37.82 mm^2^.

#### 3.2.1 Using a spectrophotometer

1. Set the wavelength at 538 nm on a spectrophotometer such as Genesys 10S UV-VIS spectrophotometer (Thermo Scientific, Waltham, MA, USA).
2. Make a blank measurement using dH_2_O.
3. Make appropriate sample dilution (5-15-fold) using dH_2_O and calculate the final DF. If the samples are diluted 5-fold, then the final DF is 50 because a 10 × ES was used per mg tissue (w/v) during the extraction step.
4. Transfer 1 mL of sample to a 1 mL cuvette and measure the absorbance.
5. Calculate betalain content as follows:

$$\begin{array}{c} Betalain\;Content\;\left(mg/kg\;fresh\;tissue\right)\\ =[(A\times DF\times 550\times 1,000)/(60,000\times 1)] \end{array}$$

#### 3.2.2 Using a 96-well plate reader

1. Set the wavelength at 538 nm on a plate reader such as a 96-well plate reader SpectraMax^®^ iD3 Multi-Mode Microplate Reader (Molecular Devices, LLC, CA, USA).
2. Make appropriate sample dilution (3-5-fold) using dH_2_O and calculate the final DF. If the samples are diluted 3-fold, then the final DF is 30 because a 10-fold ES was used per mg tissue during the extraction step.
3. Transfer 100 µL of sample into each well with at least three technical replicates.
4. Include dH_2_O for blank measurements.
5. Measure the absorbance and calculate the betalain content as follows:

$$\begin{array}{c} Betalain\;Content\;\left(mg/kg\;fresh\;tissue\right)\\ =[(A\times DF\times 550\times 1,000)/60,000\times 0.2643)] \end{array}$$

#### 3.2.3 Betalain content measurement example:

1. Sample leaf pieces are collected and added into a pre-weighted 1.5 mL microcentrifuge tube (1000 mg) and weighed again (1035 mg) to calculate the sample net weight: 1035 – 1000 = 35 mg.
2. A 10 × ES (w/v) of 350 µL is pipetted into the tube and the sample leaf pieces are ground using a plastic pestle without liquid N_2_ as described in section 3.1.1B.
3. Betalain extraction is completed as described in section 3.1.1C.

##### 3.2.3.1 Absorbance measurement using a spectrophotometer

1. A sample extract is diluted 10-fold: 100 µL sample + 900 µL dH_2_O. Final DF is 10 × 10 = 100.
2. One milliliter of diluted sample is transferred to a 1 mL cuvette and the measured absorbance is 0.815.
3. The Betalain content is [(0.815 × 100 × 550 × 1,000)/(60,000 × 1)] = 747.1 mg/kg fresh tissue.

##### 3.2.3.2 Absorbance measurement using a 96-well plate reader

1. A sample extract is diluted 3-fold: 100 µL sample + 200 µL dH_2_O. Final DF is 10 × 3 = 30.
2. One hundred microliter of diluted sample is pipetted into each well of a 96-well plate and the average absorbance of three replicates is 0.710.
3. The Betalain content is [(0.710 × 30 × 550 × 1,000)/(60,000 × 0.2643)] = 738.7 mg/kg fresh tissue.

## 4 Results and discussion

**Figure 2:**
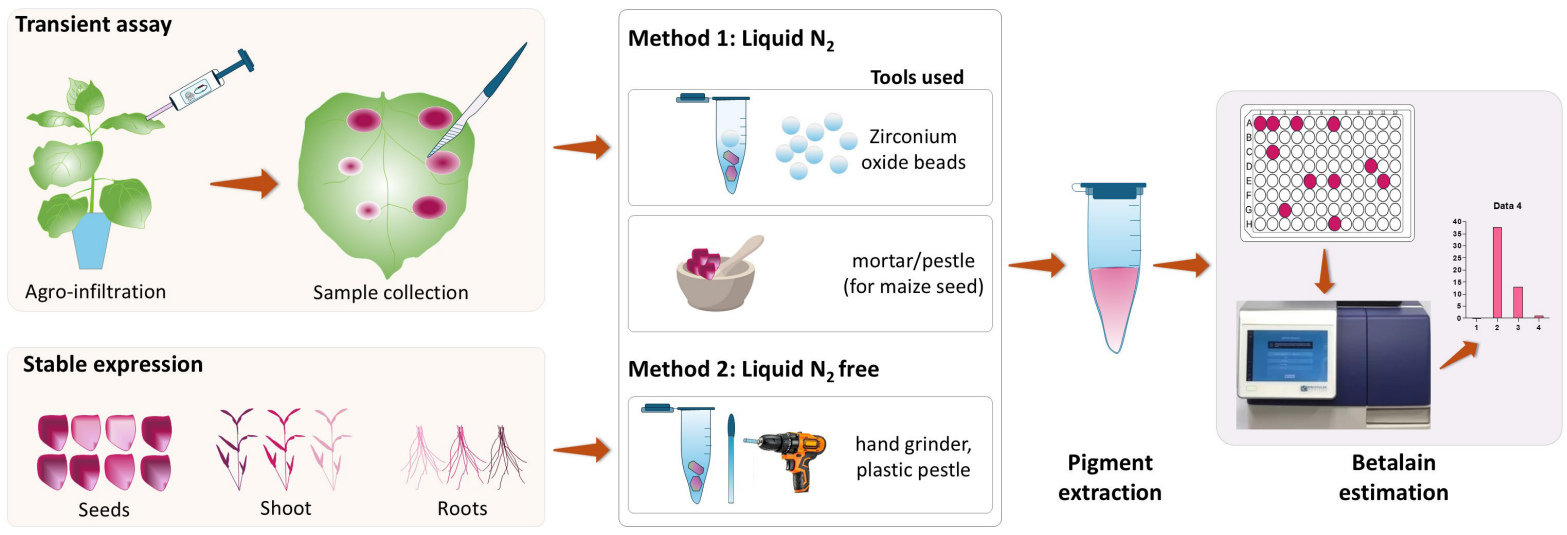
Schematic diagram showing the betalain extraction and quantification process from *RUBY*-expressing plant samples. Samples were harvested and processed using two methods. Method 1 requires sample freezing using liquid N_2_ for tissue grinding using zirconium oxide beads with an automated homogenizer or manual grinding using mortar and pestle. Ground tissue was transferred to a microcentrifuge tube for betalain extraction. The second method (Method 2) involves direct tissue grinding within a microcentrifuge tube without sample freezing using a hand grinder and a plastic pestle. For both methods, betalain was extracted using the extraction solution followed by quantification by spectrophotometric analysis (additional details in Methods).

The first step for quantifying betalain content in plant tissues is to extract the pigments. Existing protocols usually include tissue grinding in liquid nitrogen using a tissue homogenizer or mortar and pestle (Polturak et al., 2017; Grützner et al., 2021; Deng et al., 2023). In this work, we compared betalain extraction methods with and without liquid nitrogen (**Figure 2**). We demonstrated that a simple extraction method without sample freezing is a reliable option for quantifying betalain content from fresh *N. benthamiana* and maize tissue samples.

### 4.1 Betalain quantification from agroinfiltrated *N. benthemiana* leaves

We first tested the extraction of betalain from agroinfiltrated *N. benthamiana* leaves. Our primary questions were: (1) Is liquid N_2_ essential in pigment extraction, and (2) Will the extraction and measurement be consistent enough for betalain quantification, making it useful for evaluating different *Agrobacterium* strains and constructs in agroinfiltrated leaves? To answer these questions, we chose two *Agrobacterium* strains. Agro-1 is LBA4404Thy- strain harboring a ternary helper plasmid pKL2299 (Kang et al., 2022) and a T-DNA construct pCBL101-RUBY (Lee et al., 2023), whereas Agro-2 carries only pCBL101-RUBY. It is known from our previous work that the ternary *vir* helper plasmid pKL2299 enhanced plant transformation (Kang et al., 2022), therefore, they were suitable strains to test our extraction methods.

**Figure 3:**
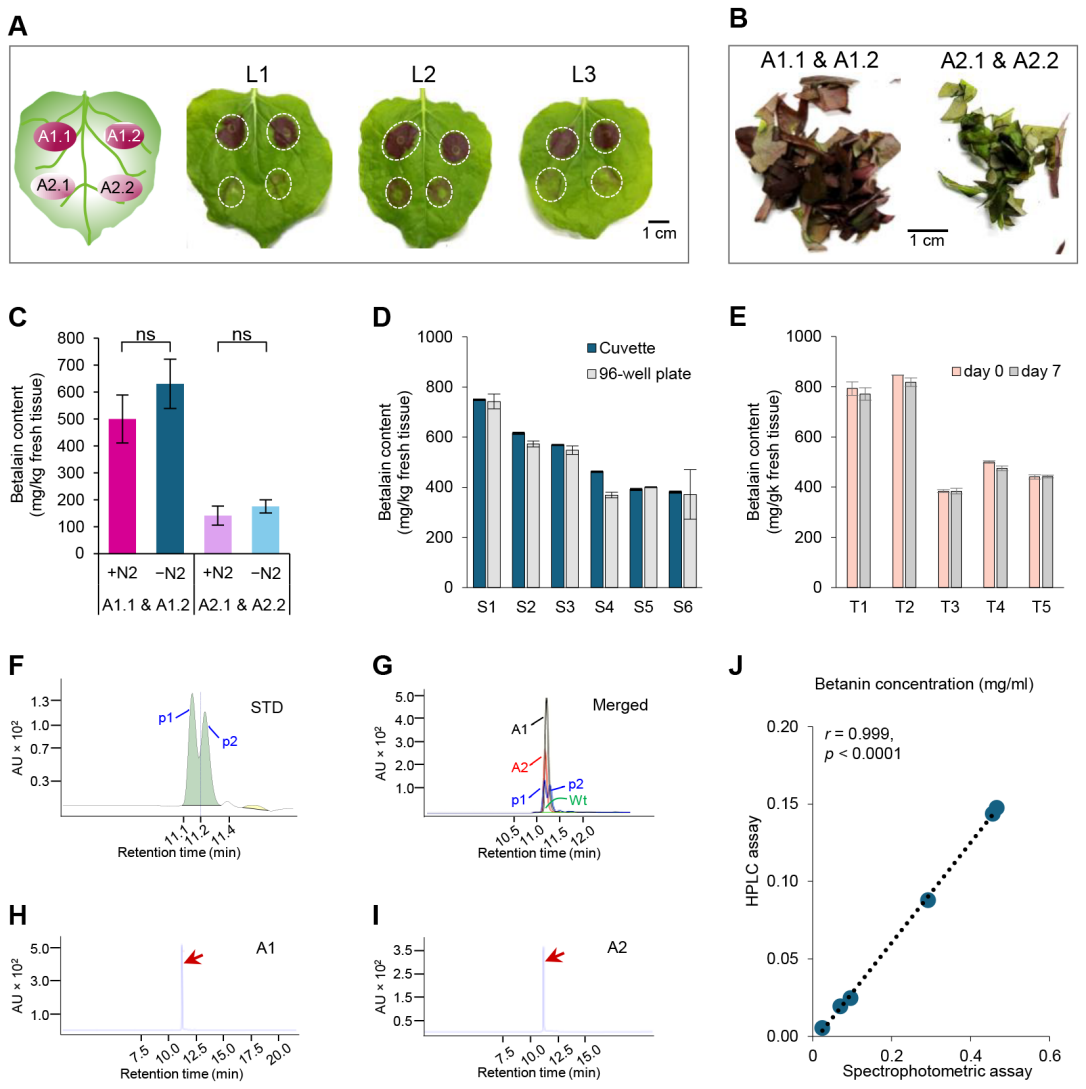
Tissue homogenization and betalain quantification methods. **(A)** *Agrobacterium*-mediated transient expression of *RUBY* in *Nicotiana benthamiana* leaves (L1 to L3). The position of infiltrated area by each strain (A1.1 & A1.2; A2.1 & A2.2) was depicted in the left carton. Leaf pictures were taken two days after infiltration. The infiltrated areas in the *N. benthamiana* leaves are marked by white dashed circles. **(B)** Infiltrated areas were cut off from the leaves and chopped into small pieces for betalain extraction. **(C)** Comparison of tissue grinding methods with or without liquid N_2_. Estimated betalain contents by different extraction methods and *Agrobacterium* strains. +N_2_, extraction with liquid N_2_; −N_2_, extraction without liquid N_2_; A1.1 & A1.2, with the ternary helper pKL2299 (Agro-1); A2.1 & A2.2, without the ternary helper pKL2299 (Agro-2). *P*-value= non-significant (ns). **(D)** Betalain quantification using two different methods: a cuvette and a spectrophotometer (Cuvette) or a 96-well plate and a plate reader (96-well plate). Betalain was extracted from 6 individual agroinfiltrated *N. benthamiana* leaves (S1-S6) without using liquid N_2_. **(E)** Analysis of betalain stability in samples (T1 to T5) extracted same day (day 0), and stored at 4 °C for seven days (day 7). n = 5 (number of independent biological replicates). **(F)** HPLC chromatogram of standard (STD) sample with two peaks (p1 and p2, separated by a dotted line with a retention time of 11.141 min) corresponding to betanin (p1, retention time = 11.1 min) and isobetanin (p2, retention time = 11.2 min), respectively. AU, Absorbance Unit. **(G)** Merged HPLC chromatogram peaks of leaf samples infiltrated by Agro-1 (A1) and Agro-2 (A2), mock control (Wt), and STD. **(H, I)** HPLC chromatograms of tested samples A1 and A2, respectively. Red arrows indicate the betanin peaks. **(J)** Scatter plot showing the correlation between the spectrophotometric and HPLC methods for betanin quantification in infiltrated *N. benthamiana* leaves (n = 6, three biological replicates for two *Agrobacterium* strains).

We infiltrated these two strains into the 4-5-week-old *N. benthamiana* leaves. Both strains were inoculated on the same leaf twice to ensure a direct comparison (**Figure 3A**, left cartoon, A1.1 & A1.2 for Agro-1, A2.1 & A2.2 for Agro-2). Two days after infiltration, purple-pigmented areas were visible. Agro-1 produced dark purple pigmentation, while Agro-2 resulted in pale purple pigmentation on the infiltrated leaf area (**Figure 3A**).

For betalain extraction, the infiltrated areas were excised from each leaf and cut into small pieces with clean scissors. Leaf pieces collected from areas infiltrated by the two strains were grouped separately (**Figure 3B**). Each group, A1.1 & A1.2 and A2.1 & A2.2, was split into equal portions to compare the two extraction methods. Previous protocols grind *N. benthamiana* leaf tissue using an automated homogenizer with liquid nitrogen (+N_2_) for betalain extraction (Grützner et al., 2021; Chang et al., 2021; Chen et al., 2023). The requirement of liquid N_2_ and tissue homogenizer often delays laboratory experiments. Hence, we were prompted to test an easy and rapid extraction method without liquid N_2_ modifying the tissue grinding step. As shown in **Figure 3C**, tissue grinding without liquid N_2_ (−N_2_) yielded slightly higher betalain content than grinding with liquid N_2_ (+N_2_) for both *Agrobacterium* strains (630 vs. 500 mg/kg tissue for A1.1 & A1.2; 175 vs. 141 mg/kg tissue for A2.1 & A2.2). Although the difference was not statistically significant (paired sample *t*-test, *P* = 0.126), slightly increased betalain content (24−25% higher than +N_2_ method) and nearly identical standard deviations of sample means (89 vs. 91 mg/kg tissue for A1.1 & A1.2; 35 vs. 24 mg/kg tissue for A2.1 & A2.2) suggest that tissue grinding in ES without liquid N_2_ using a hand grinder and a plastic pestle is an efficient method for betalain extraction from fresh leaf tissues (**Figure 3C**). For a large number of samples, a tissue homogenizer can be used to grind fresh tissue samples directly in ES.

We then compared the two absorbance measurement methods: 1) A spectrophotometer with a 1 mL cuvette, and 2) A plate reader with a 96-well plate (**Figure 3D**). Six individual agroinfiltrated *N. benthamiana* leaves were sampled and extracted for betalain quantification (S1-S6). Depending on the pigment levels, samples were diluted with dH_2_O 10-13-fold for the cuvettes and 3-5-fold for the 96-well plates, respectively. Absorbance at 538 nm was measured using both methods and the batalain content was calculated as described in the Method section 3.2. As shown in **Figure 3D**, the two methods resulted in very similar values (paired sample *t*-test, *P* = 0.125), indicating that both methods are suitable for betalain quantification. Absorbance measured with a plate reader and 96-well plates tends to have a higher variation for some samples, presumably due to pipetting errors or sample evaporation for small sample volume, and/or potential absorbance variation across the 96-well plates. Therefore, 3-4 technical replicates are recommended when 96-well plate readers are used.

Previous reports suggested that the stability of betalain can be affected by several factors such as pH, light, oxygen, metal ions, temperature, enzyme activity, and extraction solvents (Lukitasari et al., 2024). We evaluated the stability of the extracted betalain in the extraction solution. Five samples (T1 to T5) were analyzed for betalain content on the day of extraction (day 0) and after seven days (day 7) (**Figure 3E**). For storage, samples were stored at 4°C in dark conditions. We found that after one week of storage, betalain content slightly decreased (384-846 vs. 384-818 mg/kg tissue; paired sample *t*-test, *p* = 0.084; **Figure 3E**), indicating that extracted samples can be stored at 4°C for up to 7 days. Longer storage at 4°C can cause pigment degradation or evaporation of extraction solution during storage.

Betalain quantification using the spectrophotometric method (Cai and Corke, 1999; Grützner et al., 2021) is simple and rapid, thus suitable for routine comparative analysis in most laboratory settings. As a final validation step for our rapid method, we compared spectrophotometric and High-Performance Liquid Chromatography (HPLC) methods for betalain quantification. As described above, betalain samples extracted from *N. benthamiana* leaves infiltrated with two *Agrobacterium* strains (Agro-1 and Agro-2) were used for analysis with three biological replicates. As a control, *N. benthamiana* leaves infiltrated with the infiltration buffer only was included. For quantification, each sample was split into two fractions: one for the spectrophotometric measurement and the other for HPLC analysis. Spectrophotometric measurement was conducted as described above in Method section 3.2. The HPLC analysis was performed by Iowa State University W. M. Keck Metabolomics Research Laboratory. Since pure betalain is not commercially available, we used betanin (Red Beet extract diluted with Dextrin, TCI, B0397), the most abundant betacyanin produced by the *RUBY* reporter, to prepare standards for HPLC analysis. Betalain samples were separated and detected with an Agilent 1200 system equipped with a C18 column, following standard protocols (Ahmadi et al., 2020).

Standard betanin (STD) samples showed two peaks (p1 and p2) with similar retention times, indicating the presence of two betanin isomers (2S/S and 2S/R), commonly found in betalain-containing samples (**Figure 3F**) (da Silva et al., 2019; Grützner et al., 2021). The major peak corresponded to betanin (retention time = 11.1 min), followed by its isomer, isobetanin (retention time = 11.2 min). Merged HPLC chromatogram of leaf samples infiltrated by Agro-1 (A1) and Agro2 (A2), mock control (Wt), and STD, while the control sample (Wt) infiltrated with buffer only did not show any detectable peak at 538 nm (**Figure 3G**), Agro-1 (A1) and Agro-2 (A2) infiltrated samples showed a clean peak with a retention time matching the betanin standard (indicated by red arrows in **Figures 3H, I**). Betalain concentration was calculated by integrating the corresponding peak areas relative to the area of the betanin standard. As shown in **Figure 3J**, betalain contents estimated by spectrophotometric and HPLC methods were highly correlated (Pearson’s *r* (6) = 0.999, *p*< 0.0001), indicating that our rapid method is suitable for comparative analysis. As previously reported (Schwartz et al., 1981; Gonçalves et al., 2012; García-Cayuela et al., 2019), we also observed that the spectrophotometric method tends to overestimate betanin concentration compared to the HPLC method. This is because a direct absorption measurement at 538 nm by spectrophotometric method can detect impurities in the tested samples, increasing overall absorption levels.

**Figure 4:**
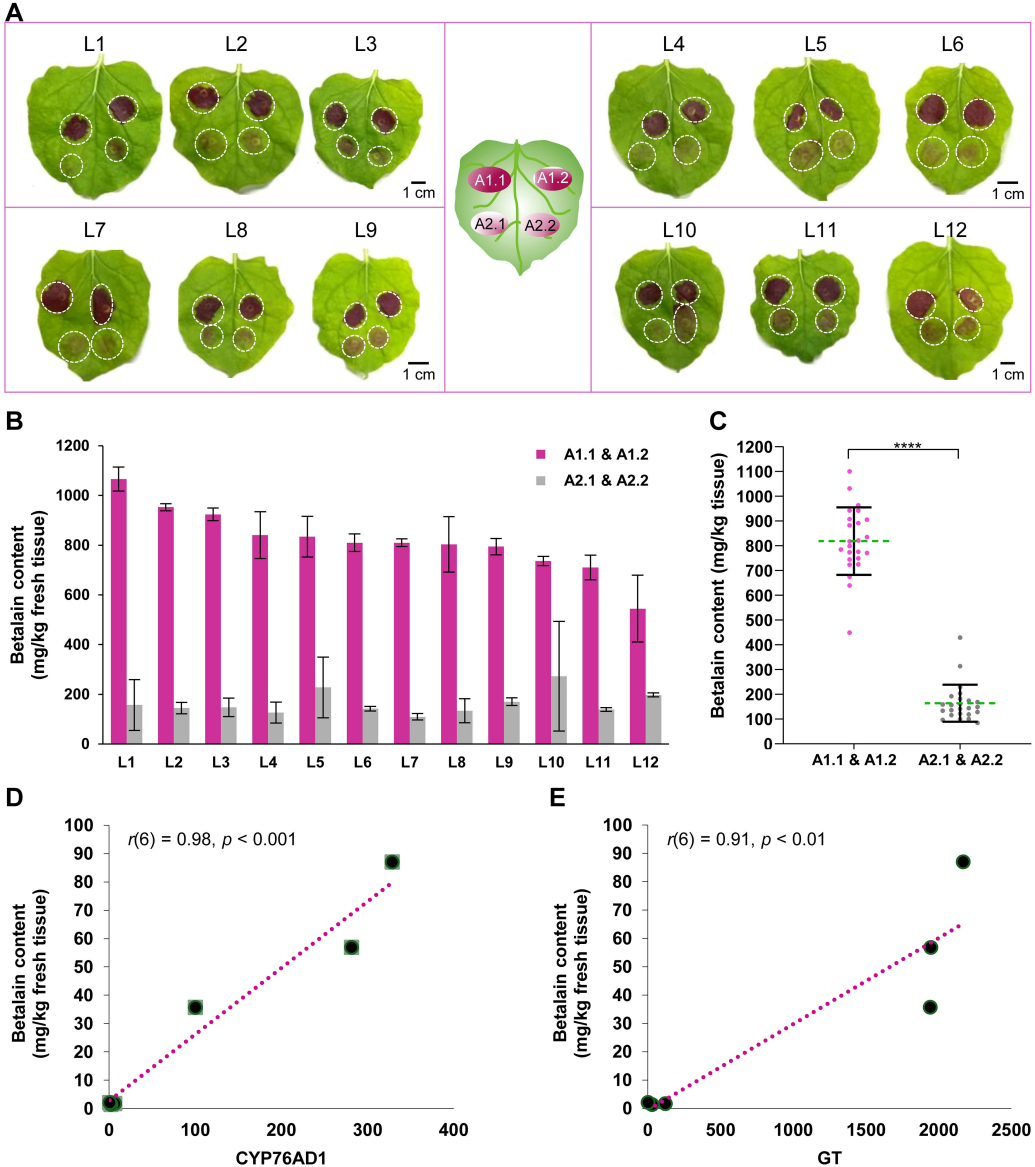
Betalain quantification from agroinfiltrated *Nicotiana benthamiana* leaves. **(A)** Leaf images were taken two days after infiltration. The center cartoon indicates the position of the infiltrated area by two *Agrobacterium* strains: A1.1 & A1.2 and A2.1 & A2.2. A total of 12 leaves (L1 to L12) were infiltrated for quantitative comparison. **(B)** Estimated betalain content from the infiltrated *N. benthamiana* leaves. Data represents the mean ± standard deviation of each leaf sample (n = 2). **(C)** Dot plots showing the distribution of betalain content by two *Agrobacterium* strains, Agro-1 (A1.1 & A1.2) and Agro-2 (A2.1 & A2.2), from 24 infiltrated sites. Each dot represents the mean value of three technical replicates of each infiltrated sample. Asterisks (****) indicate statistically significant differences (two-tailed paired sample *t*-test, *p*< 0.0001). **(D, E)** Correlation plot between *RUBY* gene (*CYP76AD1* and *GT*) expression and betalain content in infiltrated leaves (n = 6, three biological replicates for two Agro strains).

For quantitative strain comparison, 12 N*. benthamiana* leaves were infiltrated (3 leaves per experiment × 4 independent experiments) with Agro-1 and Agro-2, with two inoculations per strain on the same leaf for direct comparison (**Figure 4A**, center cartoon). For betalain quantification, agroinfiltrated areas (A1.1 & A1.2 and A2.1 & A2.2) of each leaf were collected, grouped, and analyzed individually. Betalain was extracted using our simplified protocol without liquid N_2_ and the contents in each leaf were quantified. **Figure 4B** shows that the betalain contents are consistent with the visual observation; that is, Agro-1 with the ternary helper plasmid had 2.7−7.4-fold higher pigment accumulation in *N. benthamiana* leaves than did Agro-2 (**Figure 4B**; 545−1066 vs. 110−273 mg/kg tissue) with an average of a 5-fold higher betalain content (**Figure 4C**; 819 vs. 164 mg/kg tissue). Because the transient *RUBY* expression is dependent on the number of T-DNAs delivered into the plant cells, these results suggest that the presence of extra copies of the *vir* genes on the ternary helper plasmid, strongly increases *Agrobacterium*-mediated transient transformation efficiency, as had been shown for stable plant transformation (Anand et al., 2018; Kang et al., 2022).

Furthermore, we measured and compared the *RUBY* gene expression and betalain accumulation in *N. benthamiana* leaves that were infiltrated using strains Agro-1 and Agro-2. One day post infiltration, plant samples were collected for reverse transcription-quantitative PCR (RT−qPCR) and betalain content assay. Total RNA was extracted from the leaf tissue using RNeasy Plant Mini Kit (QIAGEN, Hilden, Germany). Before cDNA synthesis, any genomic DNA contamination was removed using Turbo DNA-free DNase (ThermoFisher Scientific, MA, USA). Primers targeting the *CYP76AD1* (qPCR_F_CYP76AD1: 5′-TTCAAGCAGAACGAGCTGAC-3’; qPCR_R_CYP76AD1: 5′- CCTGCTTGATTTCCTCTTGG-3’) and *GT* (qPCR_F_GlucTF: 5′- AGGTGAAGAAACAGGGCAAG-3’, qPCR_R_GlucTF: 5′-TCTTTGGAGATCTCGCCTTC-3’) were used to estimate *RUBY* expression (Tabara et al., 2024). *NbACT2* (NbACT2_qPCR_F: 5′- TCCTGATGGGCAAGTGATTAC-3’, NbACT2_qPCR_R- 5′- TTGTATGTGGTCTCGTGGATTC-3’) was used as a reference gene (Liu et al., 2012). All the primers were synthesized by a commercial supplier Integrated DNA Technologies Inc. (IA, USA). The first strand of cDNA was synthesized from 1 µg total RNA using the M-MLV reverse transcriptase (RT) and anchored oligo-dT primer [d(T)23VN] (ProtoScript^®^ First Strand cDNA Synthesis Kit, NEB). Respective manufacturer’s guidelines were followed for any kit used in this study. RT−qPCR experiments were conducted as previously reported (Tabara et al., 2024). Betalain extraction and quantification were performed as described above. As shown in **Figures 4D, E**, betalain content was highly correlated with *RUBY* gene (*CYP76AD1* and *GT*) expression levels (CYP76AD1 vs. betalain content, Pearson *r*(6) = 0.98, *p*< 0.001; GT vs. betalain content, Pearson *r*(6) = 0.91, *p*< 0.01), indicating that quantifying betalain content is a valid method to differentiate *RUBY* expression levels in plant tissues (Tabara et al., 2024). As expected, Agro-1 exhibited significantly higher relative expression of *RUBY* genes compared to those infiltrated with Agro-2 (100-328 vs 0.8-6.3 for *CYP76AD1*; 1942-2170 vs. 3.2-121 for *GT*), confirming that the ternary *vir* helper plasmid indeed increases T-DNA delivery efficiency.

In sum, we demonstrated a simple, quick, and efficient method to extract betalain from infiltrated leaf tissues, which can be adapted for other plant species. In recent years, transient assays using agroinfiltration have been successfully tested in plants beyond the model plants, tobacco, and *N. benthamiana* (Tyurin et al., 2020; Zhang et al., 2020; Morgan et al., 2022). Thus, our simple and efficient betalain quantification method will be useful for various studies utilizing *RUBY*-reporter to evaluate transient DNA delivery methods to study gene functions or to optimize plant genetic engineering protocols.

### 4.2 Betalain quantification from transgenic maize plants

**Figure 5:**
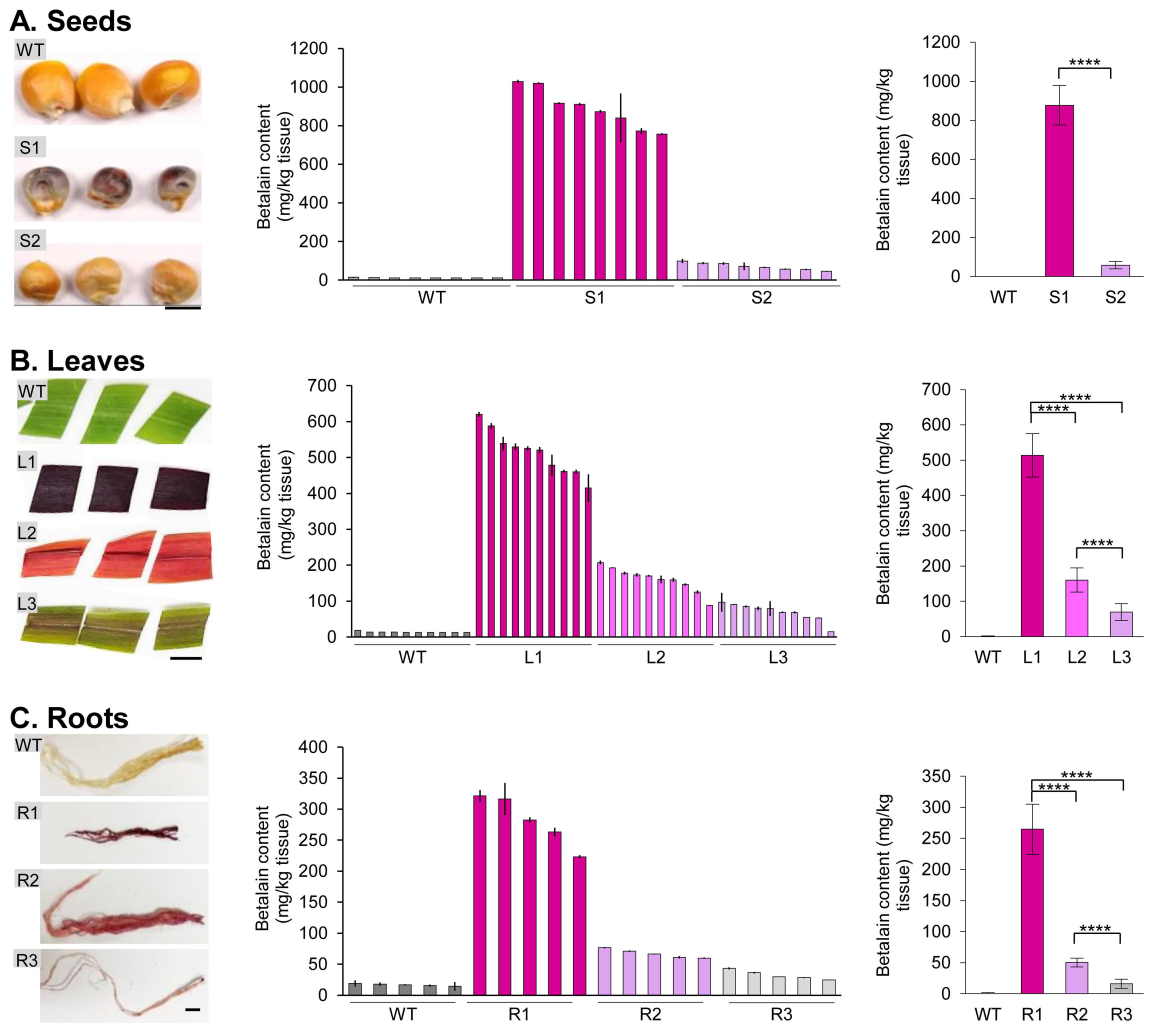
Betalain quantification from different transgenic maize tissues. **(A)** Seeds, **(B)** leaves, and **(C)** roots. Representative tissue samples were shown for each group with different betalain pigmentation levels. Wild-type maize was used as a control. Scale bar is 0.5 cm in **(A)** and 1 cm in **(B, C)**. The bar charts in the middle show the betalain content in individual plant samples. The bar charts on the right show the average betalain content (normalized with the wild type) of each group based on the visual pigmentation levels. Error bars represent the standard deviation of sample means (n = 8 for seeds; n = 10 for leaves; n = 5 for roots). Asterisks (****) indicate statistically significant differences (two-tailed paired sample *t*-test, *p*< 0.0001).

Previously, it has been observed that betalain pigmentation levels vary greatly between transgenic plants and tissue types (He et al., 2020). In stably transformed maize B104 plants, we observed differential betalain accumulation on various tissue types (Lee et al., 2023). To test if our simple extraction method can be useful for different plants and tissue types, we quantified betalain content in transgenic seed, leaf, and root tissues (**Figure 5**). Wild-type maize B104 was used as a control for this experiment.

While betalain pigmentation in transgenic seeds can be easily identified by the naked eye, some seeds with a low *RUBY* expression may not accumulate apparent phenotypes (Lee et al., 2023). We used the liquid N_2_ extraction method to grind maize seeds. The betalain contents from the wild-type maize B104 (WT) and transgenic seeds with high (S1) and low (S2) levels of pigmentation were quantified (**Figure 5A**, left panel). For each group, 8 seeds were selected and assessed independently for betalain quantification (**Figure 5A**, center panel). As can be seen, quantitative betalain content was consistent with seed colors. WT seeds showed negligible levels of betalain content of 11.9−14.8 mg/kg tissue, whereas S1 seeds accumulated the highest betalain content of 757.1−1028.7 mg/kg tissue, and S2 seeds had 45.8−98.8 mg/kg tissue. S1 seeds displayed significantly higher betalain content compared to S2 (**Figure 5A**, right panel; S1, 890.2 mg/kg tissue; S2, 71.3 mg/kg tissue; paired sample *t*-test, *p*< 0.0001).

We then germinated the transgenic seeds and quantified the betalain contents in leaf and root tissues using our simplified extraction method. Transgenic plants were divided into three groups depending on their visible pigmentation levels: dark-purple (L1 and R1, produced from line S1 seeds), light-purple (L2 and R2, same genetic background), and purple-striped (L3 and R3, same genetic background; **Figures 5B, C**, left panel). It is worth noting that these transgenic plants did not immediately correspond to the transgenic seeds described above, as the entire seed was used for betalain extraction. However, the leaf and root samples were derived from the same plant. Ten leaf samples and five root samples were collected and processed independently for betalain extraction and measurement. As with WT seeds, WT leaf tissues also showed negligible levels of betalain with 12.7−18.4 mg/kg tissue (**Figure 5B**, center panel). The betalain content in each group was consistent with their visual appearance: 414.8−620.6 mg/kg tissue for L1, 87.9−207.6 mg/kg tissue for L2, and 15.3−97.3 mg/kg tissue for L3, respectively. L1 leaf showed significantly higher betalain content compared to L2 and L3 (paired sample *t*-test, *p*< 0.0001). Whereas the betalain content of L2 was significantly higher than that of L3 (paired sample *t*-test, *p*< 0.0001).

Compared to the leaf tissue, transgenic maize plants accumulated less betalain in the roots (**Figure 5C**, center panel). On average, betalain contents in root tissues were 42−55% compared to the corresponding leaf tissues: 513.8 vs. 281.4 mg/kg tissue for L1 and R1; 160.2 vs. 67.0 mg/kg tissue for L2 and R2; 69.5 vs. 32.6 mg/kg tissue for L3 and R3, respectively (**Figures 5B, C**, right panel). Betalain content was estimated in root tissues that includes 223.4−321.5 mg/kg tissue for R1, 59.8−76.6 mg/kg tissue for R2, and 25.0−43.2 mg/kg tissue for R3, respectively. Like other tissues, WT roots had a barely detectable level of betalain, 14.3−19.1 mg/kg tissue. Among roots, R1 showed significantly higher betalain content compared to R2 and R3 (paired sample *t*-test, *p<* 0.0001). On the other hand, R2 has higher betalain accumulation than R3 (paired sample *t*-test, *p*< 0.0001). Overall, we found a clear trend of betalain contents with their visible pigmentation levels compared among tissues.

Taken together, our simple, quantitative betalain extraction and measurement method demonstrated that betalain contents in various tissues of stable transgenic maize plants were consistent with their visual appearance. It will be interesting to examine if the betalain accumulation levels are correlated with the number of transgenes in the stable events and whether betalain content can serve as a proxy for monitoring transgene expression levels, helping to identify transgenic events with optimal target gene expression.

## 5 Conclusion

The *RUBY* reporter is a highly effective visual marker for monitoring plant genetic transformation and gene expression investigations. Unlike other reporters such as GUS, GFP, and LUC, the *RUBY* system does not require costly substrates or specialized equipment to detect visible signals. Recently, *RUBY* reporters have been successfully used in many plant species, including models and crops. Its utility can be extended beyond mere visual phenotyping, as quantitative measurement of betalain content offers a valuable tool for monitoring transgene delivery and transient and stable expression in different tissues.

This study presented a simple and reliable betalain extraction and quantification method from fresh tissues of transiently transformed *N. benthamiana* and stably transformed maize plants. Importantly, our method uses a hand grinder and no liquid N_2_ for leaf tissue, which greatly reduces the laboratory resources (materials and human power) required for the process. The quantitative betalain contents matched well with the observed visual pigmentation levels in seeds, leaves, and roots. For mature maize seeds, mortar and pestle were used for grinding in liquid nitrogen. Our approach is better suited for extracting betalain from soft tissues, whereas harder tissues like seeds may necessitate the traditional extraction method for pigment retrieval. We compared the spectrophotometric and HPLC methods for betalain quantification, and there was a very strong linear correlation, indicating that while our method may not be as accurate as an HPLC-based method (Castellanos-Santiago and Yahia, 2008), our rapid protocol is a cost-effective alternative that allows quick and efficient betalain quantification for relative comparison.

In summary, the *RUBY* reporter system, coupled with our simple betalain extraction and quantification method, offers an efficient and cost-effective approach to studying gene expression in plants. This system holds significant potential for advancing plant genetic engineering, providing a valuable tool for researchers to evaluate transient DNA delivery methods, study gene functions, and optimize genetic transformation protocols.

## Data Availability

The datasets presented in this study can be found in online repositories. The names of the repository/repositories and accession number(s) can be found below: Addgene #199723, #186332.
